# Anti-bacterial antibodies in multiple myeloma patients at disease presentation, in response to therapy and in remission: implications for patient management

**DOI:** 10.1038/s41408-020-00370-7

**Published:** 2020-11-04

**Authors:** Ilaria J. Chicca, Jennifer L. J. Heaney, Gulnaz Iqbal, Janet A. Dunn, Stella Bowcock, Guy Pratt, Kwee L. Yong, Timothy D. Planche, Alex Richter, Mark T. Drayson

**Affiliations:** 1 Institute of Immunology and Immunotherapy, Clinical Immunology Service, University of Birmingham, Birmingham, UK; 2 Warwick Clinical Trial Unit, University of Warwick, Coventry, UK; 3 King’s College Hospital NHS Trust, London, UK; 4 University Hospital Birmingham NHS Trust, Birmingham, UK; 5 Department of Haematology, UCL Cancer Institute, London, UK; 6 St. George’s Hospital, University of London, Department of Medical Microbiology, Tooting, London, UK

**Keywords:** Myeloma, Myeloma

## Abstract

Multiple myeloma (MM) is associated with increased risk of infection, but little is known regarding antibody levels against specific bacteria. We assessed levels of polyclonal immunoglobulin and antibacterial antibodies in patients recruited to the TEAMM trial, a randomised trial of antibiotic prophylaxis at the start of anti-myeloma treatment. Polyclonal IgG, IgA and IgM levels were below the reference range in 71%, 83% and 90% of 838 MM patients at diagnosis. Anti-vaccine targeted tetanus toxoid antibodies were protective in 95% of 193 healthy controls but only 41% of myeloma patients. In healthy controls, protective antibodies against 6/12 pneumococcal serotypes, haemophilus and meningococcus A were present in 67%, 41% and 56% compared to just 15%, 21% and 17% of myeloma patients. By 1 year, myeloma patients IgG levels had recovered for 57% of patients whilst the proportion with protective levels of IgG against thymus-dependent protein antigen tetanus toxoid had changed little. In contrast the proportions of patients with protective levels against thymus independent polysaccharide antigens pneumococcus, haemophilus and meningococcus had fallen from 15 to 7%, 21 to 0% and 17 to 11%. Findings highlight the need for strategies to protect patients against bacterial infections during therapy and vaccination programmes during remission.

## Introduction

Multiple myeloma (MM) arises from the malignant proliferation of a single clone of plasma cells in the bone marrow and accounts for over 10% of haematological malignancies^1–3^. With the average age at diagnosis being 70 years MM causes anaemia, skeletal fractures, renal failure and profound cellular and humoral immunodeficiency^1–3^. Advances in anti-myeloma therapy have improved overall survival (OS) to 50% at 5 years but infection contributes to death in a fifth of patients with myeloma^1,2,4–7^. The risk of infection is greatest in the first 3 months after diagnosis, with a third of patients suffering serious bacterial infection, and infection contributing to half of early mortality^8–10^. The most common causes of infection are encapsulated extracellular bacteria such as *Streptococcus pneumoniae* and *Haemophilus influenza*^8^. Although novel agents and intensive therapeutic strategies have improved survival rates for myeloma, anti-myeloma therapy may further suppress patient’s immunological functions^7^.

Humoral immunodeficiency in MM is characterised by polyclonal IgM, IgG and IgA levels below the normal range in 85% of patients (immunoparesis)^11–13^. Lower levels of polyclonal IgG and IgA have been reported to be predictive of blood stream infections in the first few months from MM diagnosis and serious infection episodes during MM remission^14,15^. However, Danish registry analysis of 2557 MM patients during 6 months from diagnosis showed tumour burden and renal impairment but not immunoparesis were risk factors for infection^16^.

The level of suppression of polyclonal immunoglobulin in patients is inversely correlated with the level of M-protein and with MM stage, indicating increased suppression is identified in more aggressive forms of disease^12,13,17^. In recent UK myeloma trials median progression-free survival (PFS) was 57%, 39% and 36% longer for patients with normal IgM, IgG and IgA levels, respectively^12^. The depth of IgM suppression, but not the depth of IgG or IgA suppression, was prognostic for survival: the most severely suppressed IgM tertile of patients OS was 0.9 years shorter than those in the top tertile, and 2.6 years shorter than OS of those with normal IgM levels

Much is known about the significance of total levels of polyclonal immunoglobulins in MM with respect to survival but as we have found they are not strongly predictive of infection^12^. To our knowledge, the significance of antibody levels specific to different bacterial antigenic targets at diagnosis and following modern MM therapies has yet to be characterised. A clear picture of patients’ immunological status may help stratify them for risk of specific infections during induction therapy and may be of prognostic value for OS and PFS. Also, understanding the timeframe of immunoglobulin recovery may have an impact on the clinical management of patients.

We assessed polyclonal immunoglobulin levels and those directed against pertinent bacterial antigens at disease presentation and after 1 year in a large patient population from the UK Tackling-Early-Morbidity-and-Mortality-in-Myeloma (TEAMM) trial that had been designed to investigate the effect of antibiotic prophylaxis on infections and deaths^18^.

## Methods

### TEAMM Trial and patients

The TEAMM trial was a multicentre randomised, double-blind, placebo-controlled trial in newly diagnosed myeloma patients randomised to receive Levofloxacin or placebo for 12 weeks at the start of anti-myeloma treatment. Follow-up was 4-weekly to 16 weeks and again at 1 year. The primary outcome was time to first febrile episode or death in the first 12 weeks from start of trial treatment. Secondary outcomes included number of infections, deaths, healthcare-associated organism carriage and invasive infections, and OS^18^.

The TEAMM trial recruited between August 2012 and April 2016, 977 patients aged ≥21 years with newly diagnosed symptomatic myeloma, and within 14 days of starting active myeloma treatment. Treatment allocation was blinded and balanced by centre, estimated glomerular filtration rate (eGFR) and intention to give high-dose chemotherapy with autologous stem cell transplant. The 838 included in the present investigation out of 977 patients in the trial represent patients with sufficient serum sample available for analysis. Details of treatment, however, are limited to intended treatments at baseline as the trial did not record treatment delivered, with the exception of steroid usage. The trial (ISRCTN registration No. 51731976) had approval from the UK Coventry & Warwickshire Multi-Research Ethics Committee and all patients provided written informed consent. Serum samples from 193 healthy adult donors for determining adult ranges for the assays were from NHS Blood and Transplant, Birmingham, UK (NHSBT; age range 17–65 years).

### Laboratory analysis

Serum from diagnosis was analysed centrally at the Clinical Immunology Service (Birmingham, UK) by protein electrophoresis, densitometry and immunofixation for M-protein quantification and characterisation. Serum IgG, IgA and IgM, and kappa and lambda free light chains (sFLC) levels were quantified by turbidimetry. Patients were characterised as having one of the following multiple myelomas: IgG, IgA, IgM, IgD, light chain only (LCO), non-secretory (NS) or oligosecretory; with either kappa or lambda monoclonal light chain. Patients were classified as being below, within or above normal range (NR) for polyclonal immunoglobulins based upon 5th–95th centile ranges of adults aged over 45 years in the UK reported by Protein Reference Units: IgG 6–16 g/l; IgA 0.8–4 g/l; and IgM 0.5–2 g/l.

The serum from 838 MM patients (aged 35–90) enrolled in the TEAMM trial was analysed at disease presentation and after 1 year from first trial visit. The concentration of 19 anti-bacterial antibodies were quantified using a multiplexed Luminex assay, described in detail previously^19^. IgG antibody levels were measured against 12 pneumococcal (Pn) serotypes, 4 meningococcal (Men) serotypes, *Haemophilus influenzae* type b (Hib) polysaccharide, and tetanus and diphtheria toxoids. Protective thresholds for IgG were: 0.35 μg/ml for Pn serotypes^20^, 2 μg/ml for Men serotypes^21^, 1 μg/ml for Hib polysaccharide^22^, and 0.1 IU/ml for diphtheria and tetanus^23^. *Streptococcus pneumoniae* capsular polysaccharides were obtained from the American Type Culture Collection (Manassas, Virginia, USA), Tetanus toxoid was obtained from Quadratech (Epsom, UK), *Neisseria meningitidis* and Hib capsular polysaccharides, and diphtheria toxoid were from the National Institute for Biological Standards and Control (Potters Bar, UK). Briefly, polysaccharide antigens were pre-conjugated to poly-l-lysine (Sigma-Aldrich, Darmstadt, Germany) and purified. Poly-l-lysine-conjugated antigens and toxoids were conjugated to carboxylated microspheres (Bio-plex Systems, BioRad Laboratories, Hercules, California, USA), each corresponding to a different bead region, through a two-steps carbodiimide reaction. Beads were resuspended in phosphate-buffered saline (PBS) 0.1% Bovine Serum Albumin (BSA), 0.05% sodium azide, counted with a haemocytometer and stored in the dark at 4 °C. The analysis of serum samples for 19 antigens (19-plex) on multi-beads was performed in a 96-well filter plate. A 12 point standard curve was obtained following a 12-fold 1:2 dilution of standard serum 007 in PBS 1% BSA 0.05% Tween 20 containing 5 µg/ml pneumococcal cell wall polysaccharide (Statens Serum Institute, Copenhagen, Denmark). Serum samples were diluted 1:100 using PBS 1% BSA 0.05% Tween containing 5 µg/ml pneumococcal cell wall polysaccharide and 5 µg/ml pneumococcal serotype 22 F to prevent unspecific binding. Human serum, pooled serum and serum deprived of IgGs were used as controls. In all, 200 µl of PBS 0.05% Tween 20 were aspirated through the filter of each well to pre-wet the filter. Twenty-five microlitres of bead-conjugated antibody mix were added to each well and washed twice with PBS 0.05% Tween 20. Twenty-five microlitres of test samples were added to the plate and incubated at room temperature covered from light for 1 h on an orbital shaker at 500 rpm. After washing, 100 µl of mouse anti-human IgG phycoerythrin-conjugated antibody (Southern Biotech, Birmingham, Alabama, USA) diluted 1:200 were added and left in incubation at room temperature covered from light for 30 min on an orbital shaker at 500 rpm. Wells were washed twice and beads were resuspended in 125 µl PBS 0.05% Tween 20 for the reading. Fluorescent intensity was measured with a Luminex-200 instrument (BioRad Laboratories) and data analysis was performed with Bio-plex Manager 6.1 software (BioRad Laboratories). Polyclonal IgG, IgA and IgM were quantified by turbidimetry. Antibody levels in patients with MM were compared to a cohort of 193 healthy adult blood transfusion service donors^19^.

### Statistical analysis

Statistical analyses were performed using Prism v. 8 software (GraphPad, San Diego, California, USA) and SAS® (version 9.3; SAS Institute Inc., Cary, NC, USA) software. Statistical significance of the results was determined using the Chi-Square test to identify differences in categorical variables between groups, Wilcoxon’s rank test to compared differences between paired data and the Mann–Whitney test or Kruskal–Wallis test to analyse differences between unpaired data. Correlations between biomarkers and outcomes measures were investigated using Spearman’s rank test. Infection rates and febrile episodes were investigated based on severity of polyclonal immunoglobulin suppression. Patients were split into groups based on normal, slightly reduced and severely reduced polyclonal immunoglobulins. Incidence of infection (yes/no) or febrile episode (yes/no) in the first 12 weeks was compared between groups polyclonal immunoglobulin groups using Fisher’s exact test.

Analysis of time to first infection within 12 weeks was carried out using a log-rank comparison, starting from the date the patient started trial treatment to the date of infection, or to a censor date for those with no infections. Cox regression analyses were performed to determine independent predictors of febrile episode or death. Patient and disease characteristics were considered, including polyclonal IgM, polysaccharide antigens, tetanus and diphtheria toxoids. Overall survival was calculated from date of starting trial treatment to the date of death or censor date for those alive up to 12 months.

In addition to TEAMM treatment allocation (Levofloxacin or placebo), prophylactic Septrin was evaluated as an independent predictor of febrile episode or death. Patients were ineligible for the TEAMM trial if receiving other prophylactic antibiotic treatment, excluding pneumocystis prophylaxis if regarded as essential. Prophylactic co-trimoxazole 960 mg (Septrin) three times per week to prevent pneumocysistis was allowed and given to 314 patients within the TEAMM trial due to its wide use in the UK but was not part of stratification or randomisation. Results of the TEAMM trial showed a benefit in reducing febrile episodes for patients taking septrin prophylaxis, a benefit for levofloxacin prophylaxis and the greatest benefit for patients who took both antibiotics as prophylaxis compared to the population receiving no antibiotic prophylaxis.

## Results

### Patient characteristics

At entry to the trial patients were stratified according to intention to treat intensively with an autologous stem cell rescue procedure (transplant eligible TE) or for older frailer patients to receive non-intensive therapy (Transplant non-eligible TNE)^24^. Patient characteristics are presented in Table 1 split by intention to give intensive treatment (456 TE patients) or not (380 TNE patients). TE patients were younger, had better performance status, renal function and earlier stage myeloma disease. There was no difference in neutrophil counts with 9% having levels <1.8 × 10^9^/l but lymphopaenia (<1.2 × 10^9^/l) was commoner in TNE patients. 99% of all patients treatment included an immunomodulatory drug or proteasome inhibitor as anti-myeloma therapy and bisphosphonates.

**Table 1:** Patient characteristics by intention to give intensive treatment.

		Intention to give intensive treatment					
		No *n* = 380		Yes *n* = 456		Total *n* = 836	
Factor	Grouping	*N*	%	*N*	%	*N*	%
Age (years)	Median (IQR)	75 (70–79)	61 (54–66)	67 (60–75)			
Gender	Male	236	62	293	64	529	63
	Female	144	38	163	36	307	37
Ethnicity	White	356	94	412	90	768	92
	Mixed	1	<1	2	<1	3	<1
	Asian or British Asian	3	1	17	4	20	2
	Black or Black British	20	5	23	5	43	5
	Chinese or Other	0	0	1	<1	3	<1
	Missing	0	0	1	<1	1	<1
Performance	0	91	24	201	44	292	35
status at	1	170	45	169	37	339	41
Randomisation	2	80	21	54	12	134	16
	3	27	7	24	5	51	6
	4	1	<1	3	1	4	<1
	Missing	11	3	5	1	16	2
International Staging	Stage I	64	17	121	26	185	22
System	Stage II	117	31	182	40	299	36
	Stage III	125	33	99	22	224	27
	Missing	74	19	54	12	128	15
eGFR	>50 ml/min	246	65	381	84	627	75
	20–50 ml/min	101	26	64	14	165	20
	<20 ml/min	33	9	11	2	44	5
Prior infection	Clostridium-Difficile	2	1	1	<1	3	<1
	MRSA	6	2	5	1	11	1
	ESBL coliforms	2	1	6	1	8	1
Anti-infectives	No	237	63	323	71	560	67
in month prior	Yes	62	16	72	16	134	16
	Missing	81	21	61	13	142	17
Steroids 14 days prior to randomisation	Yes	180	47	243	53	423	51
Corticosteroids	Prednisolone	23	6	11	2	34	4
	Dexamethasone	158	42	234	51	392	47
	Other	1	<1	1	<1	2	<1
Planned anti-myeloma	Thalidomide based	185	49	178	39	363	43
treatment	Bortezomib based	120	32	146	32	266	32
	Lenalidomide based	64	17	57	13	121	14
	Lenalidomide & Carfilzomib based	3	1	71	16	74	9
	Other	8	2	4	1	12	1
Bisphosphonate	Not given	65	17	39	9	104	12
Status at	Given/will be given	313	82	414	91	727	87
Randomisation	Missing	2	1	3	1	5	1
Bisphosphonate	Zolendronate	194	51	293	64	487	58
	Pamidronate	94	25	96	21	190	23
	Clodronate	19	5	14	3	33	4
	Other	3	1	5	1	8	1
	Missing	70	18	48	11	118	14
Prophylactic anti-	No	209	55	205	45	414	50
viral/fungal	Yes	171	45	251	55	422	50
Corrected calcium	<2.5	273	72	317	70	590	70
(μmol/l)	2.5–2.75	78	20	97	21	175	21
	>2.75	15	4	33	7	48	6
	Missing	14	4	9	2	23	3
Evidence of bone disease	Yes	265	70	336	74	601	72
Site of bone disease	Vertebral fracture/collapse	115	30	114	25	229	27
	Lytic lesions	180	47	238	52	418	50
	Fractured rib	22	6	27	6	49	6
	Osteoporosis	38	10	23	5	61	7
	Other fracture	28	7	44	10	72	9
Sb2m (mg/l)	≤4	111	29	216	47	327	39
	4–8	119	31	139	30	258	31
	>8	76	20	48	11	124	15
	Missing	74	20	53	12	127	15
Anaemia (Hb g/dl)	<7.5	11	3	9	2	20	2
	7.5–10	144	38	137	30	281	34
	>10	221	58	310	68	531	64
	Missing	4	1	0	0	4	<1
Thrombocytopenia	≤150	63	17	62	14	125	15
(platelets x10^9^/l)	>150	313	82	393	86	706	84
	Missing	4	1	1	<1	5	1
Neutrophils (x10^9^/l)	<1.8	36	9	39	9	75	9
	1.8 to 3	106	28	132	29	238	28
	>3	232	61	284	62	516	62
	Missing	6	2	1	<1	7	1
Lymphocytes	<1.2	104	27	104	23	208	25
	1.2–1.8	252	66	336	74	588	70
	>1.8	18	5	15	3	33	4
	Missing	6	2	1	<1	7	1
Serum creatinine	<130	268	71	392	86	660	79
(μmol/l)	130–199	61	16	34	7	95	11
	>199	46	12	27	6	73	9
	Missing	5	1	3	1	8	1

### Polyclonal immunoglobulin levels are low at MM diagnosis and recover significantly by 1 year

**Fig. 1:**
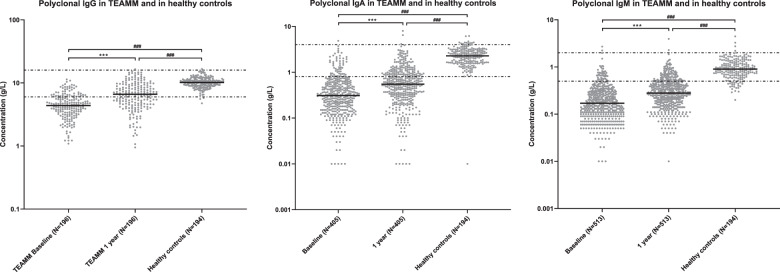
Levels of polyclonal IgG, IgA and IgM in healthy controls and TEAMM patients without an IgG, IgA and IgM M-protein, respectively, at diagnosis and 1 year post-diagnosis. Distribution of polyclonal antibody quantified in a healthy cohort and TEAMM patients without an IgG, IgA and IgM M-protein, respectively, at disease presentation (baseline) and at 1 year post diagnosis. The median line for each cohort is shown in black while the 5th and 95th centile of polyclonal normal range (IgG: 6 g/l and 16 g/l; IgA: 0.8 g/l and 4 g/l; IgM: 0.5 g/l and 2 g/l) are represented as discontinuous lines. Statistical significance was calculated using the Wilcoxon and the Mann–Whitney tests and is shown for each pair of data (***p < 0.001, ^**###**^*p* < 0.001).

Serum polyclonal IgG, IgA and IgM were measured in TEAMM patients without an IgG, IgA and IgM M-protein, respectively, at disease diagnosis and at 1 year. Polyclonal IgG, IgA and IgM levels were below the reference range (RR) in 71%, 83 and 90% of 838 MM patients at diagnosis (Fig. 1). In 196 patients with paired polyclonal IgG levels at baseline and 1 year the proportion of patients with levels within the RR increased from 25 to 57%, with median levels increasing from 4.4 to 6.6 g/l. For 405 patients with paired polyclonal IgA levels the increase by 1 year was from 15 to 33% with median levels increasing from 0.3 to 0.6 g/l. For 513 patients with paired polyclonal IgM levels at both time-points the increase in proportion with levels within the RR was from 10% at diagnosis to 21% at 1 year with median levels increasing from 0.2 to 0.3 g/l (Fig. 1). These increases in polyclonal immunoglobulin levels between diagnosis and 1 year occurred in both patients who were transplant eligible (TE) and transplant non-eligible (TNE) with no significant difference between the two cohorts (*p* = .55 for IgG, *p* = 0.57 for IgM and *p* = 0.78 for IgA (Fig. S1)). Similarly, when we split patients according to whether they were randomised to levofloxacin or placebo there was no difference in the increases in polyclonal immunoglobulin levels between diagnosis and 1 year (data not shown). For all three types of polyclonal immunoglobulins median levels in TEAMM patients at all time-points were significantly lower than median levels identified in the healthy cohort (Fig. 1).

### Severely reduced polyclonal immunoglobulins are associated with higher infection rates

Infection rates and febrile episodes were analysed according to degree polyclonal immunodeficiency (normal, slightly reduced and severely reduced) and are reported in Table S1. There was no difference in incidence of infections or febrile episodes based on degree of polyclonal IgG deficiency. Infections and febrile episodes were more likely to occur (*p* < 0.05) in patients with severely reduced polyclonal IgA (47% and 26% of patients, respectively) vs. normal polyclonal IgA (35% and 16% of patients, respectively). Similarly, incidence of infection was significantly associated with severely reduced polyclonal IgM (48%) vs. normal IgM (36%), *p* < 0.01; febrile episodes also occurred more frequently in patients with severely (23%) and slightly (22%) reduced polyclonal IgM compared to those with normal IgM levels (15%), *p* < 0.05.

### Anti-bacterial IgG antibody levels are very low at MM diagnosis

**Fig. 2:**
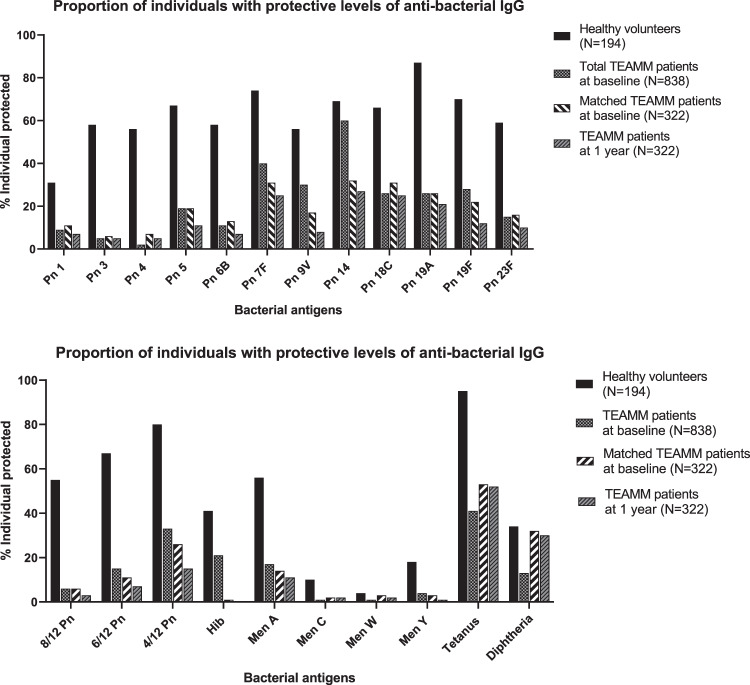
Percentage of individuals with protective levels of anti-bacterial antibodies in a healthy cohort and in TEAMM patients at disease presentation and 1 year after. Upper panel shows percentage protected for each of 12 separate pneumococcal serotypes. Lower panel shows percentages protected against at least 8/12, 6/12 and 4/12 pneumococcal serotpyes, 4 meningococcal serotypes, haemophilus, tetanus and diphtheria toxoids.

**Fig. 3:**
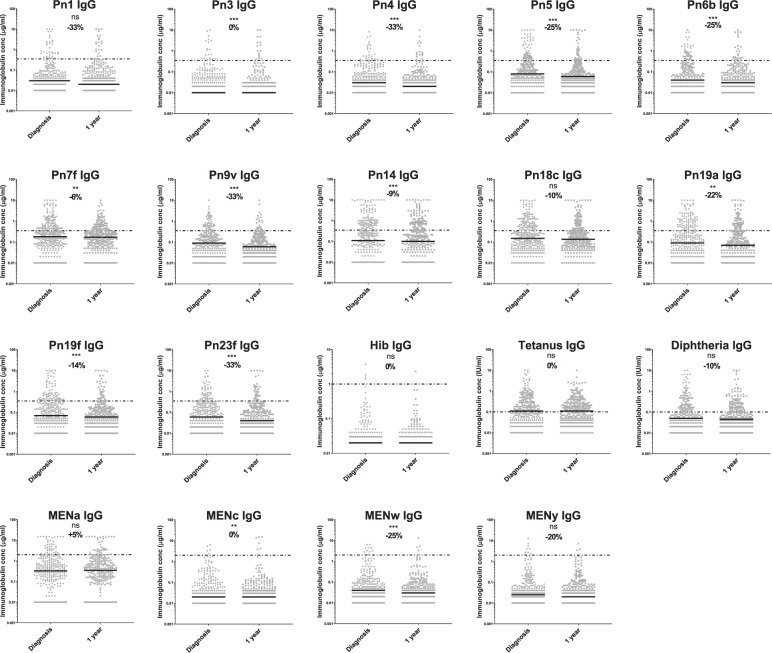
Anti-bacterial serum antibody levels in TEAMM patients at diagnosis and 1 year post-diagnosis compared with protective thresholds. Distribution of anti-bacterial antibody concentrations quantified at disease presentation (baseline) and after 1 year in TEAMM patients (*n* = 322). The median for each antibody concentration is shown in black and the recommended IgG concentrations for protection against each bacteria is presented as discontinuous lines: 0.35 μg/ml for pneumococcal (Pn) polysaccharide, 2.0 μg/ml for meningococcal (Men) polysaccharide, 1.0 μg/ml for *Haemophilus influenzae* b (Hib), 0.1 IU/ml for tetanus and diphtheria antigens. Percentage reduction between median levels at presentation and at 1 year is shown. Statistical significance was calculated using the Wilcoxon test and is shown for each pair of data (*p < 0.5; **p < 0.01; ***p < 0.001; ns *p* < 0.5).

**Fig. 4:**
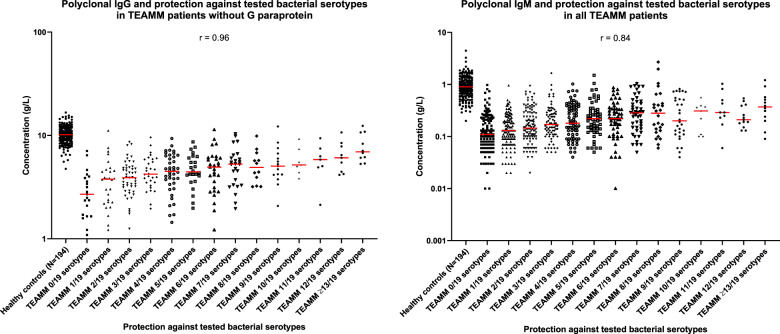
Correlation between polyclonal IgG and IgM with protection against bacterial serotype in TEAMM patients at presentation and including healthy controls for reference levels. Polyclonal IgG and IgM in a healthy cohort and in TEAMM patients in correlation with their level of protection against bacterial serotypes. Median levels of polyclonal immunoglobulins are shown in red for each group. Spearman correlation coefficient is reported for each analysis.

At diagnosis median levels of total polyclonal IgG are 4.4 g/l (43%) compared to 10.2 g/l for healthy controls. Figure 2 shows the proportions of heathy volunteers and myeloma patients that have IgG antibody levels above protective thresholds for 19 bacterial antigen targets (12 pneumococcal serotypes, 4 meningococcal serotypes, haemophilus, tetanus and diphtheria toxoids). Figure 3 shows the specific antibody levels for these 838 patients. The mean percentage of the 838 MM patients at diagnosis with protective IgG antibody levels against any one of the 19 bacterial antigen targets was 20% compared to 55% of the 193 healthy controls. Of the pneumococcal serotypes, Pn1, 3, and 4 were the most suppressed with <10% of myeloma patients showing the minimum level required for protection. The only serotype with adequate protection was Pn14, with 60% of patients achieving the threshold of ≥0.35 µg/ml and the only bacterial antigen out of all 19 investigated with more than half of patients protected. Only 6% of myeloma patients had protective levels for 2/3rd of pneumococcal serotypes compared with 55% of healthy donors. Protective levels for half and a third of pneumococcal serotypes, respectively, were 15% and 33% in myeloma patients compared to 6%7 and 80% for healthy controls. The highest level of protection for the healthy donors was found for tetanus toxoid, where 95% of individuals displayed protective levels in contrast to 41% of myeloma patients. A positive correlation was found between levels of polyclonal IgG (*r* = 0.96) and IgM (*r* = 0.84) at presentation and the numbers of bacterial serotypes for which specific antibody levels were protective (Fig. 4).

Antibody protection against bacterial antigens was found to differ between patients under 65 and greater or equal to 65 years old (Table 2). A higher proportion of younger myeloma patients had protective levels against Haemophilus, Meningococcal serotypes, Tetanus and Diphtheria toxoids. Conversely, a higher proportion of older patients had protective levels against pneumococcal serotypes.

**Table 2:** Percentage of individuals with protective levels of anti-bacterial antibodies for TEAMM patients < or ≥ 65 years old.

	Proportion of individuals with protective levels of anti-bacterial IgGs	
Antigens (protective threshold)	MM patients Under 65 (*n* = 335)	MM patients 65 and over (*n* = 503)
Pn 1 (≥0.35 µg/ml)	4.6	12.4****
Pn 3 (≥0.35 µg/ml)	*4.6*	4.1
Pn 4 (≥0.35 µg/ml)	0.9	*2.4*
Pn 5 (≥0.35 µg/ml)	14.0	23.0**
Pn 6b (≥0.35 µg/ml)	9.7	*11.5*
Pn 7 F (≥0.35 µg/ml)	38.3	*40.6*
Pn 9 V (≥0.35 µg/ml)	27.4	*31.4*
Pn 14 (≥0.35 µg/ml)	58.9	*62.3*
Pn 18 C (≥0.35 µg/ml)	17.1	31.5****
Pn 19 A (≥0.35 µg/ml)	22.6	*27.8*
Pn 19 F (≥0.35 µg/ml)	24.6	*29.3*
Pn 23 F (≥0.35 µg/ml)	13.1	*16.0*
8/12 Pn Serotypes	4.6	*6.5*
6/12 Pn Serotypes	10.9	18.4**
4/12 Pn Serotypes	28.6	*34.8*
Hib (≥1 µg/ml)	*22.0*	21.0
Tetanus (≥0.1 IU/ml)	*46.0*	36.7**
Diphtheria (≥0.1 IU/ml)	*13.7*	13.0
Men A (≥2 µg/ml)	*17.7*	16.9
Men C (≥2 µg/ml)	*1.4*	0.2*
Men W (≥2 µg/ml)	*1.7*	1.1
Men Y (≥2 µg/ml)	*4.9*	4.3

The italicised values are used to indicate higher percentages of individual with protective levels between the two age groups. Statistical significance was calculated using the Chi-square test and is shown for < 65 vs. ≥ 65 years old myeloma patients (^*^p < 0.05; ^**^p < 0.01; ^***^p < 0.001).

### By 1 year IgG antibodies against protein antigens have changed little and against bacterial polysaccharide antigens have reduced to lower levels than seen at diagnosis

Anti-bacterial antibodies were measured in paired samples from diagnosis and at 1 year for 322 MM patients. The concentration of antibodies against the 19 bacterial antigens are shown in Fig. 3 and comprise the two thymus-dependent protein antigens tetanus and diphtheria toxoids and the seventeen thymus independent polysaccharide antigens, haemophilus, 4 meningococcal serotypes and 12 pneumococcal serotypes. For the two protein antigens there was no significant difference in overall concentrations of IgG anti-toxoid antibodies between baseline and 1 year (Fig. 3), although antibody median titres were significantly increased at 1 year compared to baseline for both toxins when patients were divided by treatment in TE and TNE (30% in TNE and 63% in TE for tetanus and 100% in TNE and 33% in TE for diphtheria (Fig. S2)). No significant differences were identified in the levels of anti-toxoid antibodies at baseline between TE and TNE (*p* = 0.17 for tetanus and *p* = 0.11 for diphtheria), while anti-tetanus levels were 44% higher at 1 year in TE patients compared to TNE (Fig. S2). There was no difference in the proportions of patients protected at baseline and 1 year for tetanus (53 and 52%) or diphtheria (32 and 30%) toxins (Fig. 2). In contrast levels of antibody against polysaccharide antigens fell by a year both as median titres (Fig. 3) and proportion of patients protected (Fig. 2). For haemophilus and meningococcal C, W and Y serotypes most patients had antibody levels 50–100 times lower than the protective threshold and there was little difference between diagnosis and a year. For meningococcal A and the pneumococcal serotypes there was a reduction in antibodies between diagnosis and a year. There were significant reductions in IgG titres between diagnosis and a year for pneumococcal serotypes 3, 4, 5, 6b, 7f, 9v, 14, 19a, 19f, and 23f (Fig. 3). The percentage of people protected against 4/12 serotypes reduced from 26 to 15 and for meningococcal A from 14 to 11%. No significant differences were found between levofloxacin treatment or placebo for antibody levels or percentage protected at 1 year.

### Prediction of time to febrile episode or death within 12 weeks of starting trial treatment

No relationships were found between levels of protection against the 19 bacterial serotypes and the occurrence of deaths, febrile infections, non-febrile infections or total infections in the first 12 weeks from entry to the trial (Table S2). Cox regression analysis showed that use of prophylactic septrin was the single most important predicator of time to febrile episode or death within 12 weeks of starting trial treatment. Septrin retained significance in a multivariate model adjusting for baseline factors and antigens. A cox regression model excluding septrin identified ECOG performance status as the only factor with borderline significance (Table S3). Neither polyclonal IgM, nor the levels of specific antibody against the 17 polysaccharide and two protein bacterial targets were significant predicators of febrile episode or death (Table S3).

## Discussion

Polyclonal serum IgG, IgA and IgM levels were low at myeloma diagnosis as we and others have found previously^11–13^. This immunoparesis is present in 85% of patients and is severe. In our previous study we found immunoparesis, particularly of IgM, associated with adverse PFS and OS but not strongly associated with infection and this has been reported by other groups^12,13,25^. In the TEAMM study, infection was a major outcome in the first 12 weeks from diagnosis when infection is commonest and so this provided a good platform for more detailed investigation of the utility of polyclonal immunoglobulin levels for predicting risk of infection. Further, for the first time, we have been able to investigate, in a large number of MM patients, specific titres of antibody against a range of bacterial antigens (functional antibody) to predict infection and guide use of management strategies including prophylactic antibiotics. We show functional antibodies are even more severely compromised at disease presentation than total polyclonal antibody levels although the two are correlated.

Levels of functional antibody against the thymus-dependent antigen tetanus toxoid were the least impaired with 41% of patients protected vs. 95% of healthy controls. Following pneumococcal vaccination achievement of protective levels of antibody against two thirds of serotypes tested is considered a satisfactory antibody response. Most of the healthy donors would not have received a pneumococcal vaccination but by natural exposure to pneumococci 55% had protective antibody levels against 8/12 serotypes vs. just 6% of the myeloma patients.

The proportion of myeloma patients with protective antibody levels against tetanus toxoid was significantly lower in older myeloma patients as expected because of immunosenesence. Conversely, a higher proportion of older patients had protective levels against pneumococcal serotypes. This may reflect the UK 23-valent polysaccharide pneumococcal vaccination (PPV23) programme for individuals aged above 65^26^. Although these findings provide some support for the efficacy of the current vaccination programme in this age/patient population, significant differences were only found for 4 pneumococcal serotypes. Further, the majority of older patients still failed to meet protective levels and only 6.5% of older myeloma patients demonstrated protective levels for 8/12 or more serotypes. Previous studies have shown reduced levels of antibody production to PPV23 in MM patients^7,27^.

Higher proportions of patients were found to experience infections and febrile episodes when polyclonal IgA and IgM were severely reduced but not IgG. However, cox regression analysis showed total polyclonal antibody levels nor titres of antibody against 19 different bacterial antigenic targets predicted infections and deaths in the first twelve weeks from diagnosis. This may reflect that stratification by immunoparesis at diagnosis is limited because the great majority of MM patients have severe immunoparesis whether assessed by total or specific functional antibody levels. Additionally, they have a broad and profound immunodeficiency encompassing reduced integrity of barriers to infection and major deficits in other components of adaptive immunity and of innate immunity. The greater importance of this much broader immunodeficiency causal to high infection rates is supported by lack of efficacy of IgG replacement therapy in newly diagnosed myeloma. In a double-blind placebo-controlled trial in 203 newly diagnosed myeloma patients infusions of placebo or 18 g human polyclonal IgG on days 1, 8, 22, 43, 64 and 85 from entry to the study did not alter the incidence or type of infections and did not alter hospital admissions or antibiotic use for infections (MacLennan ICM personal communication. A Trial of Intravenous Immunoglobulin Prophylaxis in Myelomatosis for the MRC Working Party on Leukaemia in adults. Presented to the 1993 UK annual review).

There is a gradation of severity of immunoparesis from Monoclonal Gammopathy of Undetermined Significance (MGUS) to Smouldering MM and through increasing severity of MM stages^12,17^. Accordingly as anti-myeloma therapy brings patients into remission one might expect a degree of recovery from immunoparesis particularly as current myeloma treatments induce complete responses in higher proportions of patients. Post high-dose melphalan and autologous stem cell transplant about half of myeloma patients have recovery of normal levels of polyclonal immunoglobulins and these patients compared to those with sustained immunoparesis have improved PFS and OS^28–30^. We have found the same in our TE patients and importantly also (we believe for first time) that TNE patients had a similar recovery of polyclonal immunoglobulin levels post induction treatment. This observation is limited in being made at the fixed time point of 1 year from entry to the trial and not all patients will have completed their TE or TNE treatments and reached remission. A further limitation is that the trial did not extend observations beyond a year and so we have not been able to assess recovery from immunoparesis as a prognostic factor for PFS or OS.

Despite substantial recovery of total polyclonal IgG levels at 1 year in both TE and TNE patients, median titres and percentage of patients with protective IgG levels did not increase for tetanus and diphtheria toxins. Further, levels of specific IgG against bacterial polysaccharides (12 pneumococcal serotypes, 4 meningococcal serotypes and haemophilus) had fallen from very low levels at diagnosis to levels, suggesting their immune systems had reverted to a naive state. For most patients and against most bacterial antigen targets levels of antibody were 50- to a 100-fold lower than protective levels.

The findings of this study show that functional antibody levels against certain pathogens worsens following treatment and does not mirror total immunoglobulin recovery. Presumably this reflects recovery of humoral immunity following myeloma therapy being skewed by the antigenic and cytokine environment at that time and would not favour recovery of humoral immunity to pathogens to which the individual was vaccinated against or exposed against much earlier in life.

In conclusion, these data demonstrate the need to protect patients against infections at diagnosis, during anti-myeloma therapy and importantly also during remission phases. Prophylactic antibiotics and patient education, should be considered during active disease/when patients are undergoing therapy. The TEAMM trial showed a clear benefit for levofloxacin in the first 12 weeks and further studies are needed to identify optimal prophylaxis (levofloxacin, septrin, or a combination of both) and the optimal duration for prophylaxis. Vaccination should be avoided while patients are on treatment but this study clearly demonstrates the need for vaccination following treatment: in remission despite substantial recovery of total polyclonal immunoglobulin levels patient’s humoral immune system has been rendered naive by the disease and its treatment meaning they lack protective antibody levels against most bacterial antigens.

Current data on vaccination in myeloma is limited and this study highlights the necessity to determine optimal vaccination timing and need for booster vaccinations following treatment. The European Myeloma Network currently recommendations using a broad spectrum of vaccinations to overcome severely reduced antibody titres associated with autologous and particularly allogenic transplantation^31^. However, these guidelines largely reflect findings in the general/elderly population or are based on risk of disease. Consequently there is a need to design and conduct trials to investigate optimal timing and vaccination strategies in myeloma patients, specifically in relation to bacteria (such as pneumococcal, mennigocci) and also viruses (influenza) that pose a high risk to myeloma patients. The efficacy of vaccination schedules in remission should be considered and tested for all myeloma patients both in individual patients through measurement of antibody response to vaccination and in studies comparing different vaccination schedules.

## Supplementary information

Supplemental material
