# Supplementary material for: Anti-bacterial antibodies in multiple myeloma patients at disease presentation, in response to therapy and in remission: implications for patient management
Source: Blood Cancer J. 2020 Nov 4;10(11):114. doi: 10.1038/s41408-020-00370-7 (PMC7642409; doi:10.1038/s41408-020-00370-7)
Supplement: Supplementary file 1 — Supplemental material [file 41408_2020_370_MOESM1_ESM.docx]

**Supplementary information**

**Figure S1:** Levels of polyclonal IgG, IgA and IgM in TEAMM patients TE and TNE without an IgG, IgA and IgM M-protein respectively at diagnosis and 1 year post-diagnosis.

Distribution of polyclonal antibody quantified in TEAMM patients TE (intensive treatment) and TNE (non intensive treatment) without an IgG, IgA and IgM M-protein respectively at disease presentation (baseline) and at 1 year post diagnosis. The median line for each cohort is shown in black while the 5^th^ and 95^th^ centile of polyclonal normal range (IgG: 6g/l and 16g/l; IgA: 0.8g/l and 4g/l; IgM: 0.5g/l and 2g/l) are represented as discontinuous lines. Statistical significance was calculated using the Wilcoxon and the Mann-Whitney tests test and is shown for each pair of data (****p*<0.001, ns=not significant).

**Figure S2:** Levels of anti-tetanus and anti-diphtheria in TEAMM patients TE and TNE at diagnosis and 1 year post-diagnosis.

Distribution of antibody against tetanus and diphtheria quantified in TEAMM patients TE and TNE at disease presentation (baseline) and at 1 year post diagnosis. The median line for each cohort is shown in black while the recommended concentrations for protection is presented as discontinuous lines (0.1 IU/ml). Statistical significance was calculated using the Wilcoxon and the Mann-Whitney tests and is shown for each pair of data (**p*<0.05, ***p*<0.01, ****p*<0.001, ns=not significant).

**Table S1. Infection rates based on degree of polyclonal immunoglobulin deficiency**

|  |  | **Infections % (n)** | **Febrile episodes % (n)** |
| --- | --- | --- | --- |
| Polyclonal IgG (g/L) | n |  |  |
| ≥6 | 80 | 41% (33) | 19% (15) |
| ≥4 & <6 | 108 | 41% (44) | 21% (23) |
| < 4 | 125 | 42% (53) | 19% (24) |
| Polyclonal IgA (g/L) |  |  |  |
| ≥0.4 | 255 | 35% (90) | 16% (42) |
| ≥0.2 & <0.4 | 188 | 44% (83) | 20% (37) |
| <0.2 | 188 | 47% (89) | 26% (49) |
| Polyclonal IgM (g/L) |  |  |  |
| ≥0.24 | 286 | 36% (102) | 15% (43) |
| ≥0.12 & <0.24 | 259 | 42% (109) | 22% (58) |
| <0.12 | 248 | 48% (119) | 23% (58) |

Infections episodes registered within 12 weeks. Febrile episodes were defined as a recorded temperature of 37°C or above and prescribed antibiotics

**Table S2:** Frequencies of total infections, febrile infections, non-febrile infections and deaths in association with the protection against bacterial serotypes in all TEAMM patients.

| TEAMM (N=815) |  |  |  |  |  |  |  |  |  |  |
| --- | --- | --- | --- | --- | --- | --- | --- | --- | --- | --- |
|  | 0 of 19 | 1 of 19 | 2 of 19 | 3 of 19 | 4 of 19 | 5 of 19 | 6 of 19 | 7 of 19 | 8 and 9 of 19 | ≥10 of 19 |
| N | 139 | 127 | 115 | 80 | 76 | 61 | 57 | 51 | 59 | 50 |
| Total infections | 40% | 45% | 38% | 41% | 41% | 51% | 51% | 27% | 46% | 38% |
| Febrile infections | 20% | 22% | 20% | 26% | 21% | 23% | 16% | 8% | 20% | 22% |
| Non-febrile infections | 27% | 28% | 23% | 25% | 26% | 33% | 44% | 22% | 29% | 20% |
| Deaths | 13% | 11% | 18% | 14% | 17% | 3% | 7% | 10% | 14% | 8% |
|  |  |  |  |  |  |  |  |  |  |  |

Febrile episodes were defined as a recorded temperature of 37°C or above and prescribed antibiotics. Infections episodes registered within 12 weeks and deaths at 12 months.

No relationships/trends between the occurrence total infections, febrile infections, non-febrile infections and deaths, and protection against bacterial serotypes were found, table SI.

**Table S3:** **Cox regression to predict febrile episodes or deaths**

| **Factor** | **Grouping** | **HR** | **95% CI** | | ***p*** |
| --- | --- | --- | --- | --- | --- |
| 1. **N=596, events=133** |  |  |  |  |  |
| Septrin | No, Yes | 1.9 | 1.2 | 2.8 | 0.003 |
| Protective serotypes | 0-1, 2-4, 5-19 | 0.9 | 0.7 | 1.2 | 0.53 |
| Tetanus | ≤0.02, 0.03-≤0.12, >0.12 | 1.0 | 0.8 | 1.2 | 0.86 |
| Diphtheria | ≤0.01, 0.02-≤0.03, ≥0.04 | 1.1 | 0.9 | 1.4 | 0.38 |
| Polyclonal IgM | <0.5, ≥0.5 | 1.5 | 0.8 | 3.0 | 0.24 |
| 1. **N=595, events=132** |  |  |  |  |  |
| ECOG performance status | 0-2, 3-4 | 1.7 | 1.0 | 3.0 | 0.05 |
| Protective serotypes | 0-1, 2-4, 5-19 | 0.9 | 0.7 | 1.2 | 0.42 |
| Tetanus | ≤0.02, 0.03-≤0.12, >0.12 | 1.0 | 0.8 | 1.3 | 0.84 |
| Diphtheria | ≤0.01, 0.02-≤0.03, ≥0.04 | 1.1 | 0.9 | 1.3 | 0.58 |
| Polyclonal IgM | <0.5, ≥0.5 | 1.5 | 0.7 | 2.9 | 0.27 |
